# Relative independence of upper limb position sense and reaching in children with hemiparetic perinatal stroke

**DOI:** 10.1186/s12984-021-00869-5

**Published:** 2021-05-12

**Authors:** Andrea M. Kuczynski, Adam Kirton, Jennifer A. Semrau, Sean P. Dukelow

**Affiliations:** 1 University of Calgary, 1403 29th St. NW, Foothills Medical Centre, Calgary, AB T2N 0P8 Canada; 2 Section of Neurology, Department of Pediatrics, Alberta Children’s Hospital Research Institute, Calgary, AB Canada; 3 Department of Clinical Neurosciences, Hotchkiss Brain Institute, Calgary, AB Canada; 4 Department of Kinesiology and Applied Physiology, University of Delaware, Newark, DE USA

**Keywords:** Perinatal stroke, Cerebral palsy, Position sense, Proprioception, Motor control, Reaching, Robotics

## Abstract

**Background:**

Studies using clinical measures have suggested that proprioceptive dysfunction is related to motor impairment of the upper extremity following adult stroke. We used robotic technology and clinical measures to assess the relationship between position sense and reaching with the hemiparetic upper limb in children with perinatal stroke.

**Methods:**

Prospective term-born children with magnetic resonance imaging-confirmed perinatal ischemic stroke and upper extremity deficits were recruited from a population-based cohort. Neurotypical controls were recruited from the community. Participants completed two tasks in the Kinarm robot: arm position-matching (three parameters: variability [Var_xy_], contraction/expansion [Area_xy_], systematic spatial shift [Shift_xy_]) and visually guided reaching (five parameters: posture speed [PS], reaction time [RT], initial direction error [IDE], speed maxima count [SMC], movement time [MT]). Additional clinical assessments of sensory (thumb localization test) and motor impairment (Assisting Hand Assessment, Chedoke-McMaster Stroke Assessment) were completed and compared to robotic measures.

**Results:**

Forty-eight children with stroke (26 arterial, 22 venous, mean age: 12.0 ± 4.0 years) and 145 controls (mean age: 12.8 ± 3.9 years) completed both tasks. Position-matching performance in children with stroke did not correlate with performance on the visually guided reaching task. Robotic sensory and motor measures correlated with only some clinical tests. For example, AHA scores correlated with reaction time (R = − 0.61, p < 0.001), initial direction error (R = − 0.64, p < 0.001), and movement time (R = − 0.62, p < 0.001).

**Conclusions:**

Robotic technology can quantify complex, discrete aspects of upper limb sensory and motor function in hemiparetic children. Robot-measured deficits in position sense and reaching with the contralesional limb appear to be relatively independent of each other and correlations for both with clinical measures are modest. Knowledge of the relationship between sensory and motor impairment may inform future rehabilitation strategies and improve outcomes for children with hemiparetic cerebral palsy.

## Background

The perinatal period, extending early in gestation until the 28th post-natal day, harbours one of the highest risks for ischemic stroke [1]. Perinatal ischemic stroke is a cerebrovascular injury occurring in nearly 1:1000 live births, and may result in long-term functional and neurodevelopmental deficits including hemiparetic cerebral palsy (HCP) [2]. Sensory impairment in the contralesional upper limb is common, with 37% to 90% of children with HCP [3–9] demonstrating impairments in passive motion sense [5, 7, 10, 11], kinesthesia [12–14], and tactile recognition [4, 5, 9]. Additionally, more than 80% of children with HCP experience decreased motor control and coordination, weakness, spasticity, and impaired reaching and grasping with their contralesional, stroke-affected arm [14–17]. These deficits in sensory and motor function in the affected upper limb, and impairments in sensorimotor integration impact interactions with the environment and a child’s ability to complete activities of daily living. Less is known, however, about the relationship between proprioception and motor control and how this may be altered following perinatal stroke.

Advances in robotic technology offer several advantages to measure sensory and motor abilities in stroke [18–23]. Robotic measures can capture and quantify small and large changes over time and are able to deal with the challenges in reliability previously reported in sensory measures of clinical function [24]. Our group is interested in better understanding somatosensation, particularly proprioception, and its influence on motor control. Proprioception is classically defined as the ability to sense the position (position sense) and motion (kinesthesia) of one’s body. Intact proprioception is crucial to provide feedback about one’s surroundings and to guide and refine motor behavior. We have previously used the Kinarm robot to identify significant deficits in position sense and kinesthesia in the contralesional, stroke-affected arm of children with HCP [25, 26], in adults with stroke [27–31], and following traumatic brain injury [32]. Further, we demonstrated that lesions affecting the diffusion properties of the dorsal column medial lemniscus white matter sensory tracts were highly correlated with impaired proprioception [33]. With the same robot, we used a visually guided reaching task to assess motor impairment of the contralesional and ipsilesional arms of children with HCP [34]. In this study, we found significant deficits in movement length, time, and speed in the contralesional limb of children with two types of perinatal stroke. These findings of poor motor control were highly correlated with changes to corticospinal tract connectivity [35].

Deficits in perception and sensation following stroke may impact overall disability. Sensory loss has been reported to have a significant adverse impact on upper extremity motor function [36, 37] and recovery after stroke [38]. Other studies have suggested that safety, postural stability, and motor function are negatively impacted by proprioceptive deficits [39–43], while deficits in load and grip strength are related to impaired somatosensory perception in children with HCP [41, 42]. Contrary to these findings, evidence from a relatively large study of adult patients following ischemic stroke suggests conscious proprioception and motor function are independent of each other [43] and, in fact, recovery of these two modalities do not necessarily operate in parallel and can have very different timelines [28]. As most daily activities require a combination of sensory and motor function, developing a better understanding of the relationship between sensory and motor function may advance personalized therapies and improve outcomes in children with HCP.

In this study, we aimed to evaluate the relationship between robot-quantified position sense and visually guided reaching behavior of the contralesional, stroke-affected limb in children with HCP. Based on findings in adult stroke [43], we hypothesized that position sense dysfunction would be independent of impaired motor performance in children with HCP when tested in the Kinarm.

## Methods

### Participant criteria

Children and adolescents with perinatal stroke were recruited from a population-based research cohort (Alberta Perinatal Stroke Project) [44] and included in the present study if they met the following criteria:

1. Age 6–19 years, and born at term > 36 weeks gestational age.
2. Clinical and MRI confirmation of perinatal ischemic stroke.
3. Symptomatic hemiparesis: Pediatric Stroke Outcome Measure [45] sensorimotor component > 0.5, Manual Abilities Classification System (MACS) [46] grades 1–4, and child/parent perceived functional limitations.
4. Visual acuity ≥ 20/30.

Participants were excluded if they had evidence of:

1. Multifocal stroke and/or additional neurological disorders independent from perinatal stroke.
2. Severe hemiparesis: MACS [46] grade 5, or fixed contracture.
3. Severe spasticity: Modified Ashworth Scale [47] > 3 in any muscle tested.
4. Interventions in the upper extremity including surgery, botulinum toxin treatment, constraint or brain stimulation therapy within 6 months of study participation.
5. Inability to comply with study protocol.

From the community, typically developing children were recruited and completed the same evaluations if they were 6–19 years old, and were free of neurological impairments. Written informed consent/assent was obtained from all participants. Data from the study group has been previously reported in various forms [25, 26, 33–35]. A total of 193 participants were included in the present study (Table 1). This study was approved by the institutional research ethics board.

**Table 1:** Demographic information and inclusion criteria of all study participants

	Stroke	Control
Number of participants	48	145
Age (years)	12.0 ± 4.0	12.8 ± 3.9
Sex (female, male)	17, 31	72, 72
Handedness (L, R, M)	24, 22, 2	8, 124, 12
MACS [1, 2, 3, 4, 5]	[12, 21, 0, 0, 0]^a^	–
PSOM Motor [0, 0.5, 1, 1.5, 2]	[0, 5, 16, 0, 21]^b^	–

Participant age is indicated as a mean ± standard deviation. The number of participants of each sex and with each type of handedness is shown and separated by commas. Results from the Manual Abilities Classification System (MACS) and Pediatric Stroke Outcome Measure (PSOM) are shown as the number of subjects who obtained a given score (square brackets). Abbreviations: left (L), right (R), mixed (M), Manual Abilities Classification System (MACS), Pediatric Stroke Outcome Measure (PSOM). Missing data from ^a^16, and ^b^6 participants

### Clinical assessments

Prior to completing the robotic tasks, an experienced therapist performed clinical sensory and motor assessments. With the vision of their limb occluded, clinical sensory assessments included the following:

1. *Thumb and wrist position sense:* the therapist moved the participant’s thumb up and down from a neutral position. Following a single movement in one direction, participants were asked to identify the direction of the movement. Participants were scored either 0 (unable to correctly identify position) or 1 (able to correctly identify position). Three trials were completed, and assessment was then repeated for wrist position sense.
2. *Thumb localization test (TLT):* the therapist moved the participant’s contralesional arm (non-dominant arm for controls) lateral to the midline [48]. With eyes closed, participants were asked to touch the thumb of the hand moved by the therapist with their opposite thumb and index finger. Their performance was scored on a four-point scale from 0 (no difficulty locating) to 3 (unable to locate).
3. *Stereognosis:* three standardized objects (nickel, key, and paperclip) were sequentially placed in the participant’s palm, beginning with the contralesional hand (non-dominant hand in controls). The participant was asked to identify the object and the therapist scored the response either a 0 (unable to identify), 0.5 (identified category but not object), or 1 (able to identify). The task was repeated with the opposite hand and the order of the standardized objects was pseudo-randomized.
4. *Graphesthesia:* the therapist “drew” a 3, 5, or 7 sequentially in the participant’s palm with the cap of a pen and asked them to identify the number. Participants were scored either a 0 (unable to identify) or a 1 (able to identify). This task was done bilaterally with the contralesional/non-dominant hand tested first, and the order of the numbers pseudo-randomized in the opposite hand.

Thumb and wrist position sense and TLT scores were summated for a combined total score of clinical sensory performance. Individuals were considered to pass the clinical sensory outcomes if they scored 1 in both thumb and wrist position sense, and 0 in TLT. Scores of 0 in either thumb or wrist position sense and ≥ 1 in TLT indicated failure for the purposes of our analysis.

Clinical sensory measures included assessment of visual fields via confrontation technique (scored as normal, or abnormal when hemianopsia or quadrantanopsia were identified) and the Behavioural Inattention Test (BIT) which examined visuospatial function using six conventional subtests: line bisection, line crossing, star cancellation, letter cancellation, figure and shape copying, and representational drawing. Participants received a score out of 146, with scores < 130 indicating hemispatial neglect [49].

Standardized motor assessments completed by the same trained therapist included:

1. *Muscle strength:* muscle strength of the shoulder, elbow, wrist, and finger was graded bilaterally for all participants [50]. Participants received a score ranging from 0 (no contraction) to 5 (normal strength) based on the Medical Research Council scale, with a maximum summated score of 60 per arm.
2. *Modified Ashworth Scale (MAS):* the tone of flexion and extension for the shoulders, elbows, and wrists were assessed in all participants with HCP [47]. Scores ranged from 0 (no increase in tone) to 4 (rigidity) and were summed to give one total score per arm (maximum summated score of 30 per arm).
3. *Chedoke-McMaster Stroke Assessment (CMSA):* arm and hand movements were assessed bilaterally through seven stages of movement in participants with HCP [51]. Total scores ranged from 0 (paralysis) to 7 (normal movement).
4. *Assisting Hand Assessment (AHA):* bimanual upper extremity motor function was assessed in children with HCP through 22 activities [52]. Scores were expressed as logit units, ranging from 0 (no use of the stroke-affected hand) to 100 (normal function).
5. *Melbourne Assessment Unilateral Upper Limb Function (MA):* finger dexterity and speed of movement was evaluated in the contralesional, hemiparetic limb children with HCP using 16 reaching and grasping tasks of differently sized objects [53]. Total scores ranged from 0 (unable to perform) to 100 (no difficulty).
6. *Manual Abilities Classification System (MACS):* object manipulation in age-appropriate daily activities (e.g. eating, dressing, playing, drawing or writing) was explored in the contralesional, hemiparetic limb of children with HCP. Total scores ranged from 1 (handles objects easily and successfully) to 5 (unable to handle objects). For the purposes of our analysis, participants were considered to have significant hemiparesis if they scored ≥ 2.
7. *Purdue pegboard test (PPB):* unilateral fine motor function was assessed in all participants (stroke and healthy controls). Participants were instructed to pick up one peg at a time and successively fill a sequence of holes as quickly as possible in 30 s (LaFayette Instrument Co, LaFayette, IN). Each participant completed the task twice with each hand separately, and the best score of the number of holes filled was used in analysis.
8. *Modified Edinburgh Handedness Inventory:* hand dominance was determined using 10 items (e.g. hand that holds scissors) [54] in all participants (stroke and healthy controls). Scores of + 10 were given for right arm use, while scores of − 10 were given for left arm use. Equal use of both limbs was scored 0. Completely right-hand dominant individuals scored + 100, while left-hand dominant individuals scored − 100. Participants with an overall score of 0 (ambidextrous) were excluded from this study. Participants scoring between − 50 and + 50 (with the exception of 0) were classified as mixed handedness and were categorized according to their self-reported handedness in the data analysis.

### Kinarm robotic exoskeleton

**Fig. 1:**
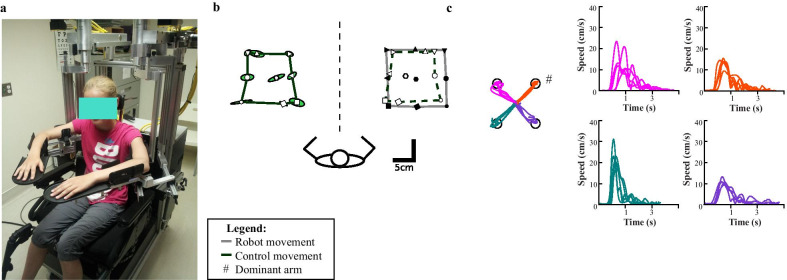
Kinarm robot sensory and motor performance. **a** A healthy adolescent is seen positioned in the wheelchair base of the Kinarm robot. Both arms are supported and rest in the troughs. **b** Position-matching performance of an 11-year-old female healthy child is shown. Black (filled) targets represent the positions where the robot moved the contralesional hand. The grey lines connect the outer eight targets for visualization purposes. Unfilled targets represent the final hand position of the ipsilesional hand. Coloured ellipses represent Var_xy_ around each target. The control participant mirror-matches the position of the contralesional, robotically moved hand with their ipsilesional hand. **c** Visually guided reaching performance of an 11-year-old healthy female is shown. The four colours indicate the trials moving in one single direction. Speed profiles for each direction of movement are shown

Robotic assessments were performed at the Foothills Medical Centre Stroke Robotics Laboratory (Calgary, AB). The Kinarm robotic exoskeleton (Kinarm, Kingston, Ontario) quantified upper limb proprioception and movement in the horizontal plane by monitoring and manipulating the shoulder and elbow joints. The Kinarm robot was modified to ensure comparable upper limb positioning of smaller participants by adding a booster seat and foam padding to the modified wheelchair base, and 2.54 cm risers to the arm troughs (Fig. 1a). Once fit in the robot with their arms supported by the exoskeleton, participants were wheeled into the virtual reality work environment where the tasks were projected onto a screen. Two tasks were utilized in the present study: arm position-matching to assess limb position sense [31], and visually guided reaching to assess unilateral motor control [27, 43]. Further descriptions of the tasks and calculations of parameters can be found in the Kinarm manual [55].

### Arm position-matching task

For participants with perinatal stroke, the robot moved the subject’s contralesional arm to one of nine spatial targets. The targets were separated by 6 cm with the 8 outer targets shaped like a square and one central target (Fig. 1b). Once the movement was complete, the participant mirror-matched the position with their opposite arm. For healthy controls, the robot moved their dominant limb and participants matched with their non-dominant arm. Each participant completed 6 blocks of trials where the order of the 9 spatial targets was pseudo-randomized for a total of 54 trials. All movements were completed with the subject’s vision of their upper extremities occluded using an opaque screen and apron. From this task, three parameters known to quantify position sense were measured [25, 28, 31, 43]. The mathematical equations for these parameters have previously been described [31] and can be found in the Kinarm manual [55]:

1. *Variability (Var*_*xy*_*):* trial-to-trial endpoint variability (in cm) of the hand position matched between the robot and participant-controlled arms.
2. *Contraction/Expansion (Area*_*xy*_*):* perceived area of the workspace the robot moved the stroke-affected limb through as indicated by the participant-controlled arm. Area_xy_ > 1 indicated expansion of the overall workspace.
3. *Systematic spatial shift (Shift*_*xy*_*):* spatial hand position difference across all targets (in cm) between the two arms.

### Visually guided reaching task

In this task, a workspace in the shape of an “X” was created with four peripheral targets arranged in the circumference of a circle 6 cm from a central target. The participant’s index finger tip was represented by a white circle (1 cm diameter). Participants were instructed to move their hand as quickly and accurately as possible from the fixed central target (red circle, 2 cm diameter) to one of the four peripheral targets (red circle, 2 cm diameter) when it was illuminated (Fig. 1c). A total of 20 trials were completed by each participant with the order of the illumination of the peripheral targets pseudo-randomized. The task was completed twice by all participants, beginning with their dominant arm. The participants were able to visualize the central and peripheral illuminated targets; however, a screen obscured their view of their upper limbs.

In this unilateral motor task, five reaching parameters quantified motor control as previously described [27, 28, 34, 43]. The mathematical equations for these parameters have been previously defined [27] and can be found in the Kinarm manual [55]:

1. *Postural speed (PS):* hand speed (in cm/s) while holding in the central target for 500 ms prior to beginning a reach to an illuminated peripheral target.
2. *Reaction time (RT):* time (in sec) from the illumination of a peripheral target to the initiation of arm movement.
3. *Initial direction error (IDE):* angular deviation (in degrees) between a) a straight line from the hand position at movement onset to the illuminated peripheral target, and b) a vector from hand position at movement onset to the position after the initial movement. The initial stage of movement was defined as the time between movement onset and the first minimum hand speed.
4. *Speed maxima count (SMC):* number of speed peaks during movement between movement onset and movement offset. SMC quantified the corrective movements.
5. *Movement time (MT):* total time (in sec) from movement onset to offset, describing the total amount of time it took the participant to complete the reaching movement from the central target to the illuminated peripheral target.

At the start of the task, the hand was held in the central target for 500 ms before a peripheral target was illuminated. During this time with the hand held in the central target, the first parameter, posture speed (PS), was calculated as a measure of fluctuations in the speed profile of the hand prior to leaving the start target [27]. We refer to the upper bound of these values (95th percentile), as PS_max_. Also during this time a second point of interest, the median value (50th percentile), was defined as PS_min_. The data from PS was then used to calculate movement onset and movement offset.

To determine movement onset, we first identified when the hand left the start target to initiate a reach to a peripheral target and then went back in time to determine when one of the following conditions was satisfied: either a) the occurrence of a local minimum in hand speed below PS_max_, or b) the point at which hand speed fell below PS_min_. This was deemed movement onset for a given reach. If the above described conditions related to movement onset were not met, or if hand speed remained above PS_max_, or if the participant took too much time to leave the central target (> 2000 ms after peripheral target illumination), movement onset was not recorded. In total, no movement onset was recorded 28 times across all trials for all individuals.

Movement offset was defined as the time when the participant reached the peripheral target and satisfied one of the following conditions: a) hand speed minima was below PS_max_, or b) a hand speed below PS_min_ was identified. If a participant did not reach the peripheral target, movement offset was not recorded and the trial was logged as having no end movement. In total, no end movement was recorded 93 times.

### Statistical analyses

SigmaPlot (Systat Software Inc., San Jose, CA, USA), SPSS (IBM, Armonk, NY, USA) and Matlab (Mathworks, Natick, MA, USA) software were used to perform statistical analyses. A one-way ANOVA with Tukey’s post-hoc test were used to compare differences in age and sex between the two groups.

In order to determine whether an individual participant failed a given parameter, we first determined the 95% range of control performance on each robotic parameter. Stroke and control participants falling outside the control range for a given parameter were classified as failing that robotic parameter. The position-matching parameter Area_xy_ required special consideration as it is a two-sided measure indicating perceived expansion and contraction of the workspace (values equalling 1 indicate exact accuracy in matching the robot area, whereas areas < 1 indicate small workspace and areas > 1 indicate larger workspace than moved by the robot). Thus, for Area_xy_ we used reciprocal values for this part of the analysis.

In order to determine whether an individual participant failed a given task, we examined the total number of parameters on each task failed by 5% of control participants. Based on healthy control performance, participants that failed ≥ 2 position-matching parameters were categorized as failing the position-matching task, while participants that failed ≥ 2 reaching parameters were categorized as failing the visually guided reaching task.

Fisher’s exact tests evaluated the relationship between proprioceptive and motor performance in the stroke group (position-matching vs. reaching: 3 parameters × 5 parameters = 15 comparisons, Bonferroni α = 0.003). Partial Spearman’s correlations controlling for age assessed the relationship between robotic proprioceptive task and motor parameters with clinical assessments (robotic task vs. clinical measure: 8 parameters × 4 clinical tests = 32 comparisons, Bonferroni α = 0.002). Fisher’s exact tests were also used to assess the relationship between clinical sensory (combined score of thumb and wrist position sense and TLT) with motor (MACS) outcomes.

## Results

A total of 193 participants were included in this study (children with HCP n = 48, controls n = 145). Age and sex were comparable among the groups (Table 1). Table 2 describes the results of the clinical assessments. Of note, all participants had full visual fields to confrontation except for two children with perinatal stroke: one had a left homonymous hemianopsia, and one did not have visual field testing completed.

**Table 2:** Clinical characteristics of study participants

	Stroke	Control		
Logit AHA [0–100]	66.9 ± 20.0 (32–100)^a^	–		
MA [0–100]	77.5 ± 20.6 (31–100)^a^	–		
BIT [0–146]	133.3 ± 18.0 (56–146)^b^	–		

	Contralesional	Ipsilesional	Non-dominant	Dominant
TLT [0, 1, 2, 3]	[32, 15, 0, 1]	[46, 2, 0, 0]	[142, 3, 0, 0]	[143, 2, 0, 0]
Position Sense [0, 1] Thumb Wrist	[12, 35]^c^ [8, 39]^c^	[4, 43]^c^ [2, 45]^c^	[1, 144] [0, 145]	[0, 145] [0, 145]
Stereognosis [0, 0.5, 1] Nickel Key Paperclip	[20, 17, 11] [24, 3, 20]^c^ [25, 1, 22]	[3, 20, 25] [6, 3, 38]^c^ [8, 4, 36]	[3, 51, 91] [4, 0, 141] [2, 0, 143]	[4, 48, 93] [6, 1, 138] [3, 1, 141]
Graphesthesia [0, 1] 7 5 3	[20, 28] [19, 29] [26, 22]	[6, 42] [6, 42] [6, 42]	[11, 134] [10, 135] [10, 135]	[9, 136] [4, 141] [12, 133]
Strength [0–60]	51.8 ± 7.4 (30–60)^c^	60.0 ± 0.3 (58–60)^c^	60.0 ± 0.09 (59–60)^d^	60.0 ± 0.2 (58–60)^c^
MAS [0–30]	2.65 ± 2.6 (0–10)	0.0 ± 0.0	–	–
CMSA Arm [1, 2, 3, 4, 5, 6, 7]	[0, 0, 14, 3, 9, 7, 15]	[0, 0, 0, 0, 0, 9, 39]	–	–
CMSA Hand [1, 2, 3, 4, 5, 6, 7]	[0, 4, 12, 5, 13, 12, 2]	[0, 0, 0, 0, 1, 13, 34]	–	–
PPB	3.81 ± 4.5 (0–14)	12.5 ± 2.1 (7–16)	13.8 ± 2.2 (8–19)	15.0 ± 2.4 (8–21)

Assisting Hand Assessment (AHA), Melbourne Assessment (MA), Behavioural Inattention Test (BIT), strength, Modified Ashworth Scale (MAS), and Purdue Pegboard (PPB) scores are shown as a mean ± standard deviation, with a range of scores shown in brackets. Results from the Thumb Localization Test (TLT), position sense, stereognosis, graphesthesia, and CMSA are shown as the number of subjects who obtained a given score (square brackets). Abbreviations: Assisting Hand Assessment (AHA), Melbourne Assessment (MA), Behavioural Inattention Test (BIT), Thumb Localization Test (TLT), Modified Ashworth Scale (MAS), Chedoke-McMaster Stroke Assessment (CMSA), Purdue Pegboard (PPB). Missing data from ^a^16, ^b^2, ^c^1, and ^d^3 participants

**Fig. 2:**
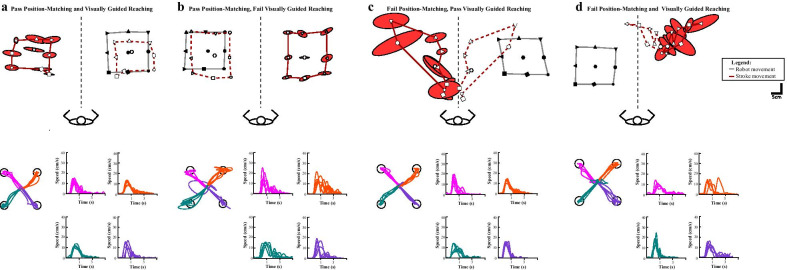
Sensory and motor performance of exemplar children with perinatal stroke. For the visually guided reaching task, each colour indicates trials moving in a single direction. Speed profiles for each direction of movement are also shown. **a** An 18-year-old female with AIS performed within normal limits of control performance on both robotic tasks. **b** A 9-year-old female with PVI performs within normal limits in position-matching, but falls outside the normal control performance in visually guided reaching. **c** A 19-year-old male with AIS falls outside the normal limits in the position-matching tasks, but performs within the normal limits in reaching. **d** A 12-year-old male with AIS performs outside the normal limits on both robotic tasks

Representative examples of the performance in each of the robotic tasks are depicted in Figs. 1 and 2 for each group (stroke and control). Assessments were well tolerated by all participants.

### Performance on Robotic Tasks

A total of 16 (33%) participants with HCP failed the position-matching task, and 24 (50%) failed the visually guided reaching task. Overall, 17 (35%) participants with HCP passed both the arm position-matching and visually guided reaching tasks. A total of 15 (31%) participants passed the position-matching task, but failed the reaching task, and 7 (15%) participants failed the position-matching task but passed the reaching task. Nine (19%) participants failed both the position-matching and reaching tasks.

**Fig. 3:**
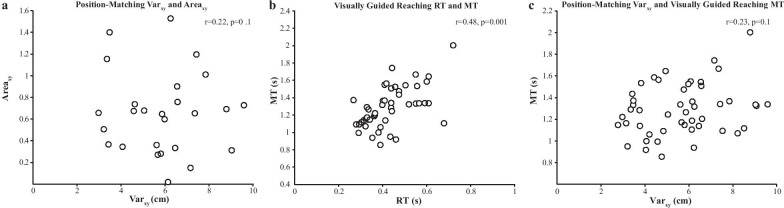
Correlations between robotic parameters. The performance of the participants with stroke is shown. **a** Position-matching Var_xy_ and Area_xy_ did not correlate (R = 0.22, p = 0.1). **b** Visually guided reaching parameters RT and MT were moderately correlated (R = 0.48, p = 0.001). **c** Position-matching Var_xy_ and visually guided reaching MT did not correlate (R = 0.23, p = 0.1)

For participants with HCP, the arm position-matching task parameters did not correlate with each other. However, within the reaching task, RT correlated with MT (R = 0.48, p = 0.001), IDE correlated with SMC (R = 0.68, p < 0.001) and MT (R = 0.68, p < 0.001), SMC correlated with MT (R = 0.74, p < 0.001) (Table 3). Fischer’s exact test between RT and MT was close to reaching statistical significance (p = 0.0056; Table 3). Comparing performance of the participants with HCP across the two robotic tasks using correlations and Fischer’s exact tests, we observed no significant relationships between the two tasks after correcting for multiple comparisons (Table 3). Interestingly, the Fischer’s exact test between position-matching Var_xy_ and visually guided reaching IDE was close to statistical significance (p = 0.0051; Table 3). Figure 3 demonstrates the relationships between selected parameters in the robotic tasks.

**Table 3:** Relationships between robotic measures in stroke subjects

		Position-matching			Visually guided reaching				
		Var_xy_	Area_xy_	Shift_xy_	PS	RT	IDE	SMC	MT
Position-matching	Var_xy_	–	0.22	0.14	0.22	0.24	0.32	0.20	0.23
	Area_xy_	0.12	–	− 0.023	0.045	− 0.050	− 0.22	− 0.15	− 0.15
	Shift_xy_	0.046	1.0	–	− 0.087	0.096	0.079	0.15	0.22
Visually guided reaching	PS	0.63	0.53	0.29	–	0.35	0.12	0.0070	− 0.21
	RT	0.74	0.66	0.46	0.22	–	0.47	0.28	0.48^*^
	IDE	5.1 × 10^–3^	0.69	0.75	0.61	0.070	–	0.68^*^	0.68^*^
	SMC	0.18	1.0	0.30	0.56	1.0	0.099	–	0.74^*^
	MT	0.38	1.0	0.52	0.62	5.6 × 10^–3^	0.014	0.049	–

In the lower left triangle, Fisher’s exact probabilities are shown. In the upper right triangle, partial Spearman’s correlation coefficients are shown. The two triangles are separated by the diagonal line of “–”. Statistical significance is denoted with * if p < 0.003 based on Bonferroni correction. Abbreviations: variability (Var_xy_), contraction/expansion (Area_xy_), systematic spatial shift (Shift_xy_), posture speed (PS), reaction time (RT), initial direction error (IDE), speed maxima count (SMC), movement time (MT)

### Clinical outcomes and relationships with robotic measures

Overall, 23 children (49%; n = 47) with perinatal stroke failed the clinical sensory test (thumb and wrist position sense, and TLT), while 20 (63%; n = 32) failed the clinical motor test (MACS). Some clinical assessment data was missing for participants resulting in a total of 31 participants with stroke having *both* MACS and clinical sensory test data available for comparison. Four children (13%) passed both the sensory and motor scores, 7 (23%) passed the sensory but failed the motor, 8 (26%) passed the motor but failed the sensory, and 12 (39%) participants failed both the sensory and motor clinical measures.

Of the 24 children that passed the clinical sensory tests, 16 (67%) also passed the robotic position-matching task. Of the 23 children that failed the clinical sensory test, 15 (65%) passed the position-matching task and 8 (35%) failed the robotic task. Eight (17%) failed both the clinical sensory test and the position-matching task. Out of the 12 children that passed the clinical motor test, only 2 (17%) passed the robotic visually guided reaching task. Conversely 3 participants (15%) that failed the clinical motor test were documented to pass the visually guided reaching task.

The clinical characteristics of children passing vs. failing robotic and clinical measures are outlined in Table 4. In participants that passed both robotic tasks (n = 17), the majority of participants had intact sensory function as measured by the clinical sensory tests. This same subset of participants had MACS scores ≤ 2, and they had the highest mean PPB scores (7.4 ± 4.7) amongst the 4 subsets. Of the 15 participants that passed the position-matching task but failed robot reaching, 6 participants (40%; n = 14 as one participant did not complete clinical position sense) had intact sensory function as per the combined clinical sensory assessment while 9 children (60%) were found to be impaired on the combined clinical sensory test. In this subset of participants, again all participants had MACS scores ≤ 2 although the majority had a score of 2 (significant hemiparesis). Of the 7 participants that failed the position-matching task but passed the robotic reaching task, 5 (71%) had intact clinical sensory function. In participants that failed both robotic tasks (n = 9), 6 participants (67%) had impaired sensory function as measured by the clinical assessments, and this subgroup had the lowest average PPB scores (1.6 ± 2.8).

**Table 4:** Clinical characteristics of participants based on robotic performance

	Pass Both Robotic Tasks	Pass PM, Fail VGR	Fail PM, Pass VGR	Fail Both Robotic Tasks
Number of participants	17	15	7	9
TLT [0, 1, 2, 3]	[12, 5, 0, 0]	[9, 5, 0, 1]	[6, 1, 0, 0]	[5, 4, 0, 0]
Position Sense [0, 1] Thumb Wrist	[2, 15] [1, 16]	[5, 9]^b^ [3, 11]^b^	[2, 5] [2, 5]	[3, 6] [2, 7]
Combined clinical sensory [0, 1]	[7, 10]	[8, 6]^b^	[2, 5]	[6, 3]
MACS [1, 2, 3, 4, 5]	[6, 5, 0, 0, 0]^a^	[2, 9, 0, 0, 0]^c^	[3, 2, 0, 0, 0]^d^	[1, 5, 0, 0, 0]^e^
PPB	7.4 ± 4.7 (0–14)	1.9 ± 2.8 (0–9)	2.3 ± 3.3 (0–10)	1.6 ± 2.8 (0–9)

Overall, 17 participants passed both robotic tasks, 15 passed PM and failed VGR, 7 failed PM and passed VGR, and 9 failed both robotic tasks. Results from the Thumb Localization Test (TLT), position sense, combined clinical sensory (TLT and wrist/thumb position sense), and Manual Abilities Classification System (MACS) are shown as the number of subjects who obtained a given score (square brackets). Purdue Pegboard scores are shown as a mean ± standard deviation, with a range of scores shown in brackets. Abbreviations: position-matching (PM), visually guided reaching (VGR), Thumb Localization Test (TL), Manual Abilities Classification System (MACS), Purdue Pegboard (PPB). Missing data from ^a^6, ^b^1, ^c^5, ^d^2, and ^e^3 participants

Clinical sensory and motor outcomes were not related (Fisher’s test, p = 0.6). Few statistically significant correlations were observed between the robotic and clinical measures (Table 5) and these were only seen when comparing a few of the visually guided reaching parameters with the clinical motor assessments (AHA, MA, and CMSA scores).

**Table 5:** Relationships between robotic and clinical measures

	Logit AHA	MA	CMSA Arm—Contralesional	CMSA Hand – Contralesional
Var_xy_	− 0.37	− 0.35	− 0.34	− 0.25
Area_xy_	− 0.092	− 0.042	0.0010	0.18
Shift_xy_	− 0.41	− 0.38	− 0.087	− 0.26
PS	− 0.075	− 0.086	− 0.049	− 0.095
RT	− 0.61^*^	− 0.47	− 0.33	− 0.51^*^
IDE	− 0.64^*^	− 0.69^*^	− 0.51^*^	− 0.41
SMC	− 0.41	− 0.47	− 0.38	− 0.33
MT	− 0.62^*^	− 0.74^*^	− 0.51^*^	− 0.55^*^

Spearman correlation coefficients are shown. Statistical significance is denoted with * if p < 0.002 based on Bonferroni correction. Abbreviations: variability (Var_xy_), contraction/expansion (Area_xy_), systematic spatial shift (Shift_xy_), posture speed (PS), reaction time (RT), initial direction error (IDE), speed maxima count (SMC), movement time (MT)

## Discussion

In this study, we assessed the relationship between position sense and motor performance of the contralesional, stroke-affected upper limb in children with HCP secondary to perinatal ischemic stroke compared to the non-dominant arm of controls. On average, children with perinatal stroke were impaired in several position-matching and visually guided reaching outcomes relative to the typically developing group, similar to our previous reports [25, 34]. Overall, 17 children passed both robotic tasks, 15 passed position-matching but failed visually guided reaching, 7 failed position-matching but passed robotic reaching, and 9 failed both robotic tasks. Our findings indicate that behavioral impairments in position sense and visually guided reaching, as tested by the Kinarm robot, can be independently observed in some participants, suggesting that sensory impairments can occur independently of motor impairment and vice versa. Our findings highlight the importance of understanding sensory and motor function and how they are related, as motor impairment is often the most salient clinical impairment seen during observations of individuals with HCP. Additionally, careful examination of motor and proprioceptive impairment will allow for identification of outcome measures to better inform rehabilitation strategies, and has the potential to reduce disability and improve functional outcomes for children with hemiparesis after perinatal stroke.

The findings of the present study are incongruent with several studies that have suggested that sensory and motor impairment are related in cerebral palsy [4, 36, 37, 41, 56–60]. These differences may be due to smaller sample sizes utilized by the aforementioned studies and the sole use of clinical assessments. It has been previously shown that clinical measures of sensory function may lack reliability and sensitivity [24] when compared to objective measurement tools, such as those provided by robotic technology. This, however, may be due to slightly different information collected by clinical and robotic measures. In the present study, we did not observe significant correlations between individual parameters in the position-matching parameters in the stroke group. Conversely, correlations were observed between individual parameters in the visually guided reaching task, specifically for the following relationships: movement time with reaction time, speed maxima count, and initial direction error; speed maxima count with initial direction error. However, we did not observe significant correlations between the position-matching and visually guided reaching parameters. Interestingly, the strongest relationship we observed, although non-significant after adjusting for multiple comparisons, was between variability in the position-matching task (Var_xy_) and initial direction error (IDE) in the reaching task. This relationship may make some sense, for example, if a participant cannot sense the location/position of their limb in space, it will be difficult to plan the trajectory and direction of the movement they intend to make.

Similar to the results of the present study, a study of 100 chronic adult stroke patients and 231 healthy controls using the Kinarm robot found that position sense and visually guided reaching function were independent for some individuals following stroke [43]. These findings are relatively surprising given the importance of proprioceptive feedback in guiding voluntary movement [61], and the plethora of studies demonstrating that proprioception is required for movement, regardless of visual input [40, 62]. For example, in order to reach for and grip an object like a juice box or hair brush, a child must be able to perceive the location of their limb relative to the size, contour, and location of that object in order to create and execute a motor plan to reach for the object with the correct positioning of the hand and fingers. It is possible that the lack of observed relationships between performance on the two robotic tasks in this study may be in part due to the position-matching task being bimanual and completed without vision compared to the unimanual reaching task with visual input [43]. It is, however, important to note that the visually guided reaching task allowed for visualization of the illuminated targets but similarly to the position-matching task, did not allow for visualization of the upper limbs. Several kinematic studies have suggested that visual information is crucial in the first stage of movement (creation of a kinematic plan) while proprioception is essential for transforming that information into neural commands that subsequently result in motion [62]. While researchers have assessed the role of visual feedback in individuals with impaired proprioception, the literature remains controversial. Studies of adults with stroke have identified that visual input was insufficient to fully compensate for impairments in position sense [63] or kinesthesia [30], two aspects of proprioception. Another study using methodology similar to the thumb localization test suggested that position sense is more accurate when vision is unreliable [64]. Moreover, studies in individuals with complex regional pain syndrome have suggested that alterations in the motor system may negatively affect proprioception and body image [65, 66]. As such, it is not well understood whether intact visual input could compensate for proprioception and improve motor control, especially in individuals following stroke where cortical visual-sensory and sensory-motor integration areas may be altered following the cerebral insult. Additionally, the position-matching task requires the individual to reflect spatial maps of limb location to the opposite side of the workspace. With this in mind, we must acknowledge the possibility that the position-matching task may require more complex and/or higher-level sensory processing when compared to the visually guided reaching task which may explain the dissociation in results we observe between the two tasks. Visual processing has been well described in two streams: one stream for perception, and the other for integrating visual information in order to guide action [67]. An alternative explanation for our results rests with the proposal that the position-matching task is assessing “sensation for perception”, whereas “sensation for action” may be more critical for the performance of reaching as tested. Thus, utilizing tasks that measured sense of motion (kinesthesia) may have demonstrated a stronger relationship with reaching.

We also examined how the robotic measures related to traditional clinical measures of sensory and motor function. Few significant correlations were found between the robotic and clinical measures in the stroke group, similar to our previous studies [25, 26, 34]. When we examined the relationships between those who pass or fail clinical and robotic measures, several inconsistencies were found between the results of the assessments (see Table 4). We suspect that this may be due to multiple factors. First, while the assessments used were generally intended to measure grossly similar constructs, the actual measurements of the tasks (e.g. position-matching task vs. TLT) are not identical and may potentially lead to discrepancies. Second, clinical sensorimotor assessments have been repeatedly criticized for lacking reliability, being insensitive to small changes in function, and having examiner bias [24, 68]. However, the implementation of both robotic and clinical tests is likely complementary, helping the examiner to more carefully characterize a given child’s impairments. Certainly, ours is not the first study to find limited relationships between robotic and clinical measures. A study of nine adult patients with chronic hemiparesis demonstrated improved motor control following three weeks of robot therapy [69]. However, these improvements on robot-measured motor control were not associated with improvements in clinical scores or function. The authors postulated that this may be secondary to robot task-specific improvements not captured by clinical assessments, and/or the inability of clinical tests to adequately capture small changes. More recently, a systematic review of robot-assisted therapies found weak to moderate correlations between robotic kinematic parameters and some clinical scores, if at all [70]. While many groups have examined changes in clinical sensory or motor function following robotic training [71–77], few have directly evaluated the relationship between changes in robot-measured function and clinical performance.

Despite these differences between the robotic and clinical measures, it is important to note that the clinical sensory and motor outcomes were also not related to each other. When assessing the clinical outcomes for participants based on their performance on the robotic tasks, the results were again relatively incongruent. For example, the majority of participants that failed the position-matching task but passed the motor reaching task were also found to pass the combined clinical sensory measure. However, in participants that passed both robotic tasks, the majority passed the combined sensory measure and had better MACS and PPB scores while the majority of participants that failed both tasks also failed the combined clinical sensory measure and had the lowest average PPB scores. Taken together, our findings seem to suggest that sensory and motor function may be impaired relatively independently of each other in children with HCP. Clinically, this raises an important point when considering treatment options.

Our study has additional limitations. First, the Kinarm robot does not allow for sensory and motor assessment of the hands and wrist, or the quantification of other aspects of movement (e.g. supination/pronation, range of motion, and force of movement), and as such a comprehensive analysis of sensory and motor function of the entire upper limb was not feasible. Furthermore, the majority of clinical sensory assessments provided information about the hand and wrist, while the robot provided information from the shoulder and elbow joints. While the clinical motor assessments examined the entire upper limb, this may limit the applicability of the sensory data. In the present study, we assessed conscious proprioception. While unconscious proprioception is also related to the control of movement [78], we are unable to test it currently, and future studies using a motor task that eliminates visual input may further elucidate whether sensory and motor function are related without vision potentially compensating for motor impairment as in our current task. Furthermore, our statistical post-hoc corrections were quite stringent which also affects the number of statistically significant results depicted in the present analysis. Like many samples of individuals with perinatal stroke, we had an equal number of left- and right-handed individuals. We made comparisons, however, to a control group that had only 10% left-handed individuals, similar to the population. While this represents the general population, comparisons of left-handed participants with perinatal stroke may be less meaningful when compared to a predominantly right-handed control group. Future studies may wish to consider enrolling an equal distribution of handedness in controls for comparisons with children with perinatal stroke.

## Conclusions

In conclusion, our findings support the idea that sensory and motor impairments can be independent. Deficits in position sense may need to be considered as functionally relevant and distinct when planning interventional strategies in individuals with perinatal stroke. Robotic quantification of sensory and motor function is feasible, objective, and well tolerated in children and may have added utility to explore complex relationships between sensory and motor functions in HCP. Our use of the Kinarm robot adds greater kinematic, objective details to better understand sensory and motor relationships.

## Data Availability

Data supporting the conclusions of this article is included within the article.

## Abbreviations

HCP: Hemiparetic cerebral palsy
Kinarm: Kinesiological Instrument for Normal and Altered Reaching Movements
Var_xy_: Variability
Area_xy_: Area contracted/expanded
Shift_xy_: Systematic spatial shift
PS_max_: Maximum posture speed
PS_min_: Minimum posture speed
PS: Posture speed
RT: Reaction time
IDE: Initial direction error
SMC: Speed maxima count
MT: Movement time
BIT: Behavioural Inattention Test
MAS: Modified Ashworth Scale
CMSA: Chedoke-McMaster Stroke Assessment
AHA: Assisting Hand Assessment
MA: Melbourne Assessment of Unilateral Upper Limb Function
PPB: Purdue pegboard
